# Age differences in the prosocial influence effect

**DOI:** 10.1111/desc.12666

**Published:** 2018-04-15

**Authors:** Lucy Foulkes, Jovita T Leung, Delia Fuhrmann, Lisa J Knoll, Sarah‐Jayne Blakemore

**Affiliations:** ^1^ UCL Institute of Cognitive Neuroscience London UK; ^2^ Department of Education University of York York UK; ^3^ MRC Cognition and Brain Sciences Unit Cambridge UK

## Abstract

Social influence occurs when an individual's thoughts or behaviours are affected by other people. There are significant age effects on susceptibility to social influence, typically a decline from childhood to adulthood. Most research has focused on negative aspects of social influence, such as peer influence on risky behaviour, particularly in adolescence. The current study investigated the impact of social influence on the reporting of prosocial behaviour (any act intended to help another person). In this study, 755 participants aged 8–59 completed a computerized task in which they rated how likely they would be to engage in a prosocial behaviour. Afterwards, they were told the average rating (in fact fictitious) that other participants had given to the same question, and then were asked to rate the same behaviour again. We found that participants' age affected the extent to which they were influenced by other people: children (8–11 years), young adolescents (12–14 years) and mid‐adolescents (15–18 years) all significantly changed their ratings, while young adults (19–25 years) and adults (26–59 years) did not. Across the three youngest age groups, children showed the most susceptibility to prosocial influence, changing their reporting of prosocial behaviour the most. The study provides evidence that younger people's increased susceptibility to social influence can have positive outcomes.

**RESEARCH HIGHLIGHTS**

- Children and adolescents were more likely than adults to change their ratings of prosocial behaviour as a result of social influence.
- Children (8–11 years old) showed the most susceptibility to prosocial influence.
- The age of the “influencer” did not affect the extent of susceptibility to prosocial influence, for any age group.
- Heightened susceptibility to social influence in young people can have positive outcomes.

## 1 INTRODUCTION

Individuals frequently change their thoughts and behaviour to align with those of other people, a process known as social influence. Previous research has shown that susceptibility to social influence is at its highest in late childhood (approximately age 8–10 years) then gradually decreases across the adolescent years (approximately 11–18 years) and into adulthood (19 years and above; Knoll, Leung, Foulkes, & Blakemore, 2017; Knoll, Magis‐Weinberg, Speekenbrink, & Blakemore, 2015; Steinberg & Monahan, 2007; Sumter, Bokhorst, Steinberg, & Westenberg, 2009). As such, relative to adults, children and adolescents are particularly susceptible to being influenced by others.

Adolescence begins with the onset of puberty and ends when an individual reaches adult independence (Blakemore & Choudhury, 2006). It is a period of significant social reorientation (van den Bos, 2013; Nelson, Leibenluft, McClure, & Pine, 2005), when individuals start to spend less time with their family and more time with their peers (Lam, McHale, & Crouter, 2014; Larson & Richards, 1991), and place more importance on what their peers think about them (O'Brien & Bierman, 1988). The heightened susceptibility to social influence in adolescents, relative to adults, paired with increased independence from their family, can lead adolescents to take more risks when with their peers. For example, adolescents take more risks in a simulated driving game when being watched by friends, whereas adults do not (Chein, Albert, O'Brien, Uckert, & Steinberg, 2011; Gardner & Steinberg, 2005). Knowing that peers are engaging in substance use (alcohol, tobacco and illicit drugs) increases the likelihood of adolescent substance use (Lundborg, 2006). Children and adolescents, compared to adults, are also more likely to change their perception of risky behaviours based on other people's perception of those risks (Knoll et al., 2015).

Heightened social influence in children and adolescents is often viewed through this prism of risky behaviour (i.e., negative or antisocial influence). However, social influence is not an inherently negative process: individuals of all ages can be influenced by others to behave in a more positive manner (Barry & Wentzel, 2006; van Hoorn, van Dijk, Meuwese, Rieffe, & Crone, 2016). *Prosocial influence* occurs when people engage in more prosocial behaviour (any act intended to help other people) as a result of seeing or learning about prosocial behaviour in others. (Prosocial behaviour may be altruistic, in which the act that helps someone occurs at the expense of the helper, but not always; e.g., Batson, 1987.)

Prosocial influence occurs across all ages. For example, learning about other people's prosocial actions was associated with an increase in donations to charity (Frey & Meier, 2004; Nook, Ong, Morelli, Mitchell, & Zaki, 2016; Shang & Croson, 2009) and an increase in fairness in economic games in adults (Peysakhovich & Rand, 2015). Adolescents aged 12 to 16 gave a more generous allocation of coins to their group after they saw peers approve such behaviour (van Hoorn, van Dijk et al., 2016), and 12‐ to 15‐year‐olds were also more likely to volunteer to help others in their community if they believed other students in their school were doing so (Choukas‐Bradley, Giletta, Cohen, & Prinstein, 2015). It has been long established that children will behave more prosocially if they see other people behaving this way, a process known as “imitative altruism” (e.g., Rushton, 1975). The prosocial behaviour does not need to be seen personally: children aged 4 to 9 shared more of their sweets in a one‐shot Dictator Game even if they are just told that other children in the game were generous (McAuliffe, Raihani, & Dunham, 2017).

Previous studies on prosocial influence have typically investigated relatively narrow age groups in isolation, leaving it unclear how this phenomenon might change across age. In particular, it is unknown whether the age effects seen in social influence on behaviours like risk‐taking—typically a decrease in susceptibility from late childhood to adulthood (Knoll et al., 2015; Steinberg & Monahan, 2007; Sumter et al., 2009)—would also be seen with prosocial influence. This would elucidate whether the increased social influence seen in children and adolescents (relative to adults) could also have positive outcomes. The current study therefore aimed to investigate prosocial influence between childhood and adulthood.

We also investigated whether the source of information—specifically, the age of the potential “influencer”—affects the extent to which a person is socially influenced. A previous study of social influence on risk perception assessed whether information from adolescents or adults had more impact on changing participants' perception of risk (Knoll et al., 2015). In this study, participants were asked to rate the riskiness of everyday scenarios. They were then shown the average rating provided by (fictitious) previous participants, either adolescents or adults, and were asked to re‐rate the same scenario. This study found that all age groups were influenced by other people's ratings, but this social influence effect decreased with age. Importantly, this study found that children (8–11 years) and adults (19–59 years) were more influenced by adults, older adolescents (15–18 years) were equally influenced by adolescents and adults, and only younger adolescents (12–14 years) were more influenced by adolescents than adults (Knoll et al., 2015). This suggests that the source of information impacts the degree of social influence differently at different ages.

With regard to prosocial influence, one study compared the impact of friends' and parents' volunteering behaviour (such as organizing an event or collecting money for charity) on adolescent participants' own volunteering (van Goethem, van Hoof, van Aken, Orobio de Castro, & Raaijmakers, 2014). Friends had a larger influence than parents on older adolescents' (16–19 years) behaviour, but an equal influence on younger adolescents' (12–15 years; van Goethem et al., 2014). Other studies found that parents and peers both influence adolescents' volunteering (Law, Shek, & Ma, 2013) and prosocial behaviour (Law et al., 2013; Masten, Juvonen, & Spatzier, 2009), but did not compare the relative extent of influence exerted by parents and peers.

The current study assessed the effect of two variables, participant age and information source (*adolescents* or *adults*), on prosocial influence in a large group of participants aged 8 to 59. Prosocial influence is measured here as the extent to which participants change reports of their own prosocial behaviour after seeing how much others endorse the same prosocial behaviour. Including a large age range allowed us to assess potential non‐linear changes in prosocial influence, such as heightened prosocial influence by peers in adolescence. To study prosocial influence, we adapted a paradigm originally designed to assess social influence on risk perception (Knoll et al., 2015). The participant is first asked to rate how likely they would be to engage in a prosocial act, such as carrying someone's bag for them or buying someone a gift (rating 1). The participant is then shown the average rating that other participants gave for the same behaviour. This rating was purported to be from either *adolescent* or *adult* participants, but all ratings were in fact fictitious. Finally, the participant is asked to re‐rate the same prosocial act (rating 2).

This design enabled us to address two questions: the effect of participant age on susceptibility to prosocial influence, and the effect of the source of information (either *adolescents* or *adults*) on prosocial influence. We had two hypotheses:

1. *Age differences in prosocial influence hypothesis*: The extent to which participants change their ratings from rating 1 to rating 2 will decrease with age. This is based on previous evidence that the magnitude of susceptibility to social influence (typically for risky or negative domains) decreases over time (Knoll et al., 2015; Steinberg & Monahan, 2007; Sumter et al., 2009).
2. *Source of prosocial influence hypothesis*: The extent of prosocial influence will be affected by the source of information (either *adolescents* or *adults*). Specifically, we hypothesized that children and adults would be more influenced by ratings provided by *adults*, mid‐adolescents (15–18 years) would be equally influenced by *adults* and *adolescents*, while only young adolescents (12–14 years) would be more influenced by ratings provided by *adolescents* (Knoll et al., 2015).

## 2 METHOD

### 2.1 Participants

Participants were visitors to the Science Museum in London, UK, between May and June 2016. A total of 828 participants were recruited via advertisements across the museum. Data from 74 participants were excluded from the analysis, either because the participant did not complete the task (*N* = 2), had a diagnosis of Autistic Spectrum Disorder (*N* = 7) or a learning disability (*N* = 4), had a friend or family member watching how they performed (*N* = 9), had difficulty reading the task in English (*N* = 36), had no age recorded (*N* = 1), or reported in the debriefing that they guessed the provided ratings were fake (*N* = 5). To match our age range with that of a previous study using a similar paradigm (8 to 59 years; Knoll et al., 2015) and facilitate comparison between the two studies, we excluded data from 10 participants who were older than 59. This left a total of 755 (445 female) participants in the final sample, aged 8 to 59 years (*M* = 23.16, *SD* = 11.46). Participants were divided into five age groups, again based on Knoll et al. (2015): children aged 8–11 years (*N* = 115; *M* = 9.50, *SD* = 1.10); young adolescents aged 12–14 (*N* = 49; *M* = 13.02, *SD* = 0.85); mid‐adolescents aged 15–18 years (*N* = 123; *M* = 16.67, *SD* = 1.07); young adults aged 19–25 years (*N* = 232; *M* = 21.23, *SD* = 1.91); and adults aged 26–59 years (*N* = 235; *M* = 37.27; *SD* = 8.87).

The study protocol was approved by the university ethics committee and by the Science Museum. All participants aged 16 and over gave informed consent prior to participation. Parental consent was obtained prior to participation for participants aged 15 and under.

### 2.2 Data collection

The study was conducted as part of a month‐long Live Science residency at the Science Museum, in which researchers give science demonstrations or collect data from museum visitors. Live Science is run in a separate area at the back of one of the Science Museum galleries. Four laptops were set up in this space, and museum visitors were offered the opportunity to take part in a psychology study about how frequently they engaged in different types of social behaviour. A team of three or four researchers was present, and each participant had the instructions explained to them verbally by one of the researchers. The laptops were sufficiently spread out so that participants could not see each other's screens, or talk to anyone else while taking part. Family and friends sat away from the laptops if they were not taking part themselves. On the rare occasion that a participant's performance was watched by other visitors, their data were removed from analysis (*N* = 9).

### 2.3 Prosocial influence task

#### 2.3.1 Stimuli

Seventy‐nine sentences describing a prosocial behaviour, such as “Raise money for charity”, were used as stimuli in the task (see Supplementary Materials for the full list). The sentences covered a wide variety of situations and all described a behaviour intended to help another person (for example, a friend, neighbour or family member) that could be carried out by anyone from age 8 upwards. The recipient of the prosocial behaviour in the scenario was often specified (e.g., “Help a family member clean their car”; “Help a friend if they have fallen”) but not always (e.g., “Give something you like to charity”). The gender of the recipient was never specified. Each participant saw a randomly selected 12 scenarios out of the possible 79, so all participants rated a range of behaviours with a range of recipients; this was because potential differences elicited by different scenarios was not a focus of this study. As an additional control for this, scenario was included in the model as a random effect.

Moderately prosocial behaviours that could reasonably elicit a variety of response ratings were chosen to ensure that the randomly generated providing rating (purportedly from other participants) would be believable across the full range of the scale.

**Figure 1:**
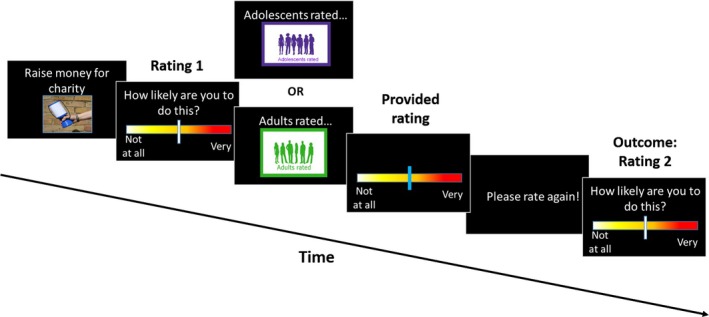
Trial sequence. First, participants were shown (for 3 s) a sentence and image that depicted a prosocial behaviour (in this example, “Raise money for charity”). They then rated how likely they would be to engage in that behaviour, using a computer mouse to move a slider on a visual analogue scale (Rating 1; no time restriction). Next, participants were shown (for 2 s) a screen saying either “Adolescents rated…” or “Adults rated…”, and were then shown a rating of the same situation, purportedly the average answer provided by a group of either adults or adolescents (2 s; this number was randomly generated). Finally, they saw a screen saying “Please rate again!” (2 s), and then were able to rate again how likely they would be to engage in that prosocial behaviour (Rating 2; no time restriction)

The stimuli consisted of a single sentence at the top of the screen with an image depicting the scenario underneath (see Figure 1). The images were included to make the task more engaging. The majority of images did not depict people (for example, the image showed a birthday card, or the outside of a train). The images that did contain people included those with a range of ages, from late childhood to adulthood.

#### 2.3.2 Trial sequence

Before the task, participants read instructions on the screen, which were also explained verbally by the experimenter. There were no practice trials. All participants were told that the *adolescents* group contained answers from people aged 12–18 years old, and the *adults* group contained answers from people aged 19–60, to ensure that everyone had the same understanding of who the groups represented.

Each participant completed 12 trials (six for each social influence group, *adolescents* and *adults*). The 12 scenarios used for each participant were randomly selected from the total of 79 available, and the order in which participants saw *adult* or *adolescent* trials was randomized. The whole task took approximately 12 minutes.

It is important to note that we did not have a control condition in this task. In a similar task that investigated social influence on risk perception (Knoll et al., 2015), there was a third condition alongside the *adult* and *adolescent* conditions. In these control trials, participants saw their own rating again (rather than seeing a rating purportedly from other participants) before being asked to rate for a second time. This condition was previously included to assess whether there were differences across age groups with respect to how much participants changed their answers from the first to the second time under no social influence. However, the previous authors found no significant differences between age groups in this condition (Knoll et al., 2015). Because of this, and to keep to the time restrictions imposed by the museum, we did not include a control condition in the current study.

The rating scale was anchored with the words “Not at all” at its leftmost point and “Very” at its rightmost point. When participants were required to make a rating, the slider first appeared at a random position on the scale in order to avoid any consistent anchoring bias. The position chosen by the participant was recorded to two decimal places (Not at all = 0.00; Very = 10.00). After the task, participants were debriefed and told that the ratings from other participants were in fact randomly generated.

The task was programmed using Cogent 2000 (University College London Laboratory of Neurobiology; http://www.vislab.ucl.ac.uk/cogent_2000.php) and run in MATLAB (version R2012b; MathWorks Inc., Natick, MA).

### 2.4 Statistical analysis

Linear mixed‐effects models were used for all analyses. All statistical analyses were conducted in R (R Core Team, 2013) using lme4 (Bates, Mächler, Bolker, & Walker, 2014) and were based on the models used by Knoll and colleagues (Knoll et al., 2015; Knoll et al., 2017). Linear mixed effects models provide appropriate estimators for unbalanced designs (Schielzeth & Nakagawa, 2013).

#### 2.4.1 Rating 1 analysis

We first ran a mixed‐effects model investigating age differences in initial prosocial ratings (rating 1). The dependent variable was rating 1 and the independent variable was age group, which was Helmert‐coded and thus followed an orthogonal coding scheme. Subject‐specific and scenario‐specific intercepts were included as random effects, which took into account individual differences in susceptibility to social influence and individual differences in social influence elicited by specific scenarios. Post‐hoc pairwise comparisons (Bonferroni‐adjusted) were run to further explore age group differences.

#### 2.4.2 Prosocial influence analysis

This analysis investigated the degree to which participants changed their prosocial ratings in the direction of the provided rating, and whether the extent of this change depended on the participant's age (*Age differences in prosocial influence hypothesis*) and/or the source of the information (*adolescents* or *adults* randomly generated provided rating; *Source of influence hypothesis*). Because the provided rating was a randomly generated number between 0.00 and 10.00, it was not related in any systematic way to rating 1.

The dependent variable in the model was the absolute difference between rating 1 and rating 2 (represented herein by *change in rating*). Independent variables in the model were the absolute difference between the provided rating and rating 1 (represented herein by *Δrating*); two‐way interactions between Δrating and age group (five levels: children, young adolescents, mid‐adolescents, young adults, adults), and Δrating and source (*adolescents*,* adults*); and a three‐way interaction between Δrating, age group and source. The variable Δrating was included in the model as a means of assessing whether the difference between the participant's rating 1 and the provided rating affected the extent to which they changed their rating.

$$\begin{array}{cc} change\;in\;rating= & \Delta rating\;+\;(\Delta rating\;\times \;age\;group)\;+\;\\ & \left(\Delta rating\;\times \;\mathrm{source})\;+\;(\Delta rating\;\times \;\mathrm{source}\;\times \;age\;group\right) \end{array}$$

Because the outcome variable and the Δrating variable were absolute values, there was no information about direction of influence in the model. This decision was made because we were not investigating the effect of the direction of influence (although this question is examined in Knoll et al., 2017); we were only interested in the magnitude of influence.

Age group and source were Helmert‐coded. (Supplementary analysis was also conducted in which age was entered as a continuous variable; see Supplementary Materials.) Two intercepts were included as random effects: subject‐specific, which took into account individual differences in susceptibility to social influence, and scenario‐specific, which took into account differences in susceptibility to social influence elicited by specific scenarios. The final model was based on 9060 observations from 755 participants.

We followed up the significant interaction between Δrating and age group by running five further models, which were identical to the model described above except that age group was dummy‐coded. For each of the five models, a different age group was used as the reference group, thereby allowing us to compare changes in ratings between all age groups. We inspected the slope of Δrating for the reference group in each model to determine whether, for each age group, Δrating was a significant predictor of the change in rating from rating 1 to rating 2 (five slopes, Bonferroni‐corrected for five tests). We also inspected the contrasts of the interaction term to see whether the extent to which age groups changed their rating differed from each other (10 contrasts, Bonferroni‐corrected for 10 comparisons).

## 3 RESULTS

### 3.1 Rating 1 analysis

We ran a linear mixed‐effects model investigating age differences in initial prosocial ratings (rating 1). The main effect of age group on prosocial ratings was significant (χ^2^ (4) = 9.71, *p* = .046), but no pairwise comparisons between age groups survived Bonferroni correction for multiple comparison (*p*s = .189–1.000; see Supplementary Table 1).

### 3.2 Prosocial influence analysis

We ran a linear mixed‐effects model to examine the extent to which participants changed their rating from rating 1 to rating 2, after seeing the provided rating purportedly from other people. We also examined whether this was influenced by participant age and/or the source of the provided rating (*adolescents* or *adults*; see Table 1).

**Table 1:** Main linear mixed‐effects model predicting change in rating

Predictor	χ^2^	*p*
Δrating	χ^2^ (1) = 62.36	< .001
Δrating × age group	χ^2^ (4) = 128.45	<.001
Δrating × source of information	χ^2^ (1) = 1.69	.194
Δrating × age group × source of information	χ^2^ (4) = .83	.829

There was a significant main effect of Δrating (see Table 1), indicating that participants demonstrated greater changes from rating 1 to rating 2 when the disparity between their rating 1 and the provided rating was greater. This suggests that participants were socially influenced by the provided rating in that they changed their own rating more when there was a greater difference between their first rating and the rating they believed came from other participants. There was a significant interaction between age group and Δrating (see Table 1), indicating that participant age affected the extent to which participants were socially influenced (*Age differences in prosocial influence hypothesis*). There was no interaction between Δrating and source type, and no three‐way interaction between Δrating, source type and age, indicating that the source of information (*adolescent* or *adult*) did not affect the extent to which participants were socially influenced (*Source of influence hypothesis*).

**Figure 2:**
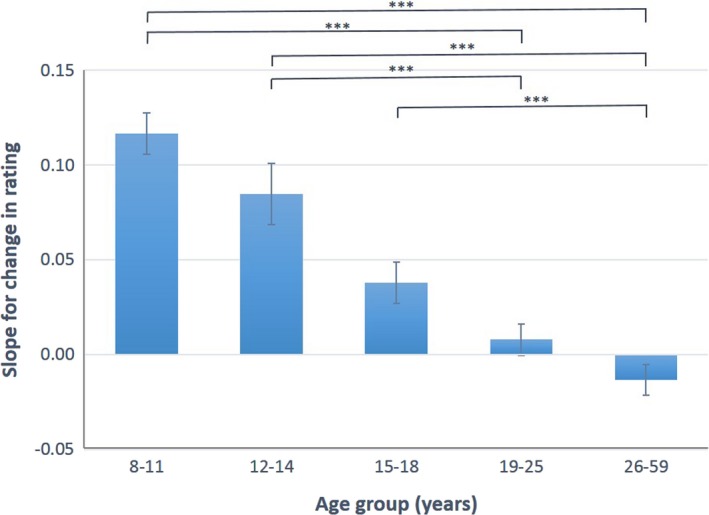
The slopes for the average change in prosocial rating predicted by the difference between the provided rating and the first rating (Δrating), shown separately for each age group. The slopes were calculated using estimates of the linear mixed‐effect models. Error bars represent standard error. ***p < .001 (Bonferroni‐corrected)

We then inspected all possible contrasts of the interaction between age group and Δrating to examine which age groups differed from one another (see Figure 2). The slope of Δrating showed that the youngest three age groups were all significantly socially influenced (i.e., they all changed their rating from rating 1 to rating 2 after seeing the provided rating; children: *t*(845) = 10.76, *p* < .001; young adolescents: *t*(835) = 5.26, *p* < .001; mid‐adolescents: *t*(923) = 3.53, *p* < .001). The slope of Δrating was not significant for either of the adult groups, indicating that they did not change their answer after seeing the provided rating (young adults: *t*(1119) = .97, *p* = 1.000; adults: *t*(1085) = −1.66, *p* = .967). Raw data plotting Δrating against the change in rating for each age group is plotted in Supplementary Figure 1.

Planned comparisons showed that children were more socially influenced than mid‐adolescents (*t*(718) = −5.45, *p* = < .001), young adults (*t*(716) = −8.57, *p* = < .001) and adults (*t*(709) = V10.33, *p* = < .001). Young adolescents were more socially influenced than young adults (*t*(763) = −4.42, *p* = < .001) and adults (*t*(773) = −5.69, *p* = < .001). Mid‐adolescents were more socially influenced than adults (*t*(740) = −4.12, *p* = < .001). All other comparisons were non‐significant (*p*s = .123–.929; see Supplementary Table 2). Therefore, there was a general decrease in susceptibility to social influence as age increased. A supplementary model that included age as a continuous variable also showed a linear decrease in susceptibility to social influence across age (see Supplementary Materials and Supplementary Figure 1).

## 4 DISCUSSION

The current study investigated the effect of age on prosocial influence, measured here as the tendency to increase reports of one's own kind, helpful behaviour as a result of seeing or hearing about this behaviour in others. We found that susceptibility to prosocial influence decreased with age. Children (8–11 years), young adolescents (12–14 years) and mid‐adolescents (15–18 years) all showed susceptibility to prosocial influence, while young adults (19–25 years) and adults (26–59 years) did not. Across the three youngest age groups, there was a decrease in the extent of prosocial influence, with children showing the most susceptibility to social influence. These findings indicate that young people's increased susceptibility to social influence can have positive outcomes, such as increasing the reporting of prosocial behaviour.

Previous studies investigating social influence in young people have often focused on negative outcomes, such as dangerous risk‐taking or antisocial behaviour. For example, studies have examined the effect of social influence on adolescents' driving risks (Chein et al., 2011; Gardner & Steinberg, 2005), substance use (Caouette & Ewing, 2017; Lundborg, 2006) and risk perception (Knoll et al., 2015). Studies that have compared age groups have found that susceptibility to these types of social influence is high in childhood and/or adolescence and then decreases with age (Chein et al., 2011; Gardner & Steinberg, 2005; Knoll et al., 2015). The current study suggests that there is a similar decrease in social influence across age with regard to positive, prosocial behaviour, indicating that young people may be especially likely to be positively socially influenced.

The results highlight the need to view social influence as an important part of social development that can have positive consequences (van Hoorn, Fuligni, Crone, & Galván, 2016). Other studies have demonstrated the benefits of this prosocial influence in real‐world settings. For example, one study investigated the efficacy of an anti‐bullying programme in which 11–16‐year‐olds were encouraged to lead a grassroots campaign to reduce student conflict in their school (Paluck, Shepherd, & Aronow, 2016). Compared with control schools, in which no special anti‐bullying programmes were introduced, student conflict was reduced by 30%. In addition, when the anti‐bullying campaign was led by more popular students it had a greater positive effect on behaviour (Paluck et al., 2016). Another study found similar effects in children aged 5–7 using a classroom behaviour management programme called the Good Behaviour Game (GBG; Dolan et al., 1993). In this programme, children were divided into teams and given rewards (e.g., stickers) if all members of the team refrained from antisocial, disruptive behaviour. The study found that children who took part in the GBG showed significantly reduced levels of aggressive behaviour (Dolan et al., 1993). These studies indicate that children and adolescents can influence one another to behave in a more positive, socially acceptable manner. The current study extends the existing literature by assessing prosocial influence in a large group of individuals across a wide age range (8–59 years), enabling us to better understand how age affects prosocial influence across the lifespan.

There was an interesting difference between the current results and the previous study using a similar methodology to investigate social influence on risk perception (Knoll et al., 2015). The previous study found that the source of influence (*adolescents* or *adults*) affected the extent to which participants were socially influenced: children (8–11 years), young adults (19–25 years) and adults (26–59 years) were more influenced by information coming from *adults* than *adolescents*; mid‐adolescents (15–18 years) were equally influenced by both groups, and only young adolescents (12–14 years) were more influenced by *adolescents* than by *adults*. In the current study, there was no effect of the source of information on prosocial influence in any age group.

It is unclear why the source of the information might be more important with regard to risk perception than reports of prosocial behaviour. One possibility is that differences in the specific paradigm used might be associated with different effects of social influence sources. Specifically, Knoll et al. (2015) asked participants about their risk perception of a given behaviour (“How risky do you think [this behaviour] is?”), while in the current study, participants were asked how likely they were to engage in a behaviour (“How likely would you be to do [this behaviour]?”). This difference in the question might for some reason be associated with differing degrees of social influence. This could be evaluated in future studies that systematically manipulate the specific question being asked. A second possibility is that people may have stereotypical beliefs about how certain age groups perceive risks (e.g., “*Adolescents perceive behaviours as less risky than adults*”), but not have any particular beliefs about how age affects prosocial behaviour, and so in the prosocial task the specific source of information (*adolescents* or *adults*) had less of an effect. Future research should investigate this possibility by assessing stereotypical beliefs alongside measures of prosocial influence.

In the current study, only the youngest three groups (children, young adolescents and mid‐adolescents) showed susceptibility to prosocial influence; young adults (19–25 years) and adults (26–59 years) did not. This is in contrast with previous studies, which have shown that adult participants increase their prosocial behaviour when learning about this behaviour in others (Frey & Meier, 2004; Nook et al., 2016; Shang & Croson, 2009). One possible explanation is the difference in type of prosocial behaviour measured. Previous studies have often focused on monetary measures such as charity donations (Frey & Meier, 2004; Nook et al., 2016; Shang & Croson, 2009) or fairness in economic games (Peysakhovich & Rand, 2015). The current study used a broader range of prosocial behaviours, such as caring for someone who is ill or giving someone directions, and perhaps these behaviours are less susceptible to social influence in adults. Secondly, our study asked about hypothetical behaviour, whereas previous studies have measured actual behaviour (e.g., amount of money donated), and it may be that the latter is more likely to be affected by social influence. These are speculative possibilities, which require further investigation.

There are a number of limitations of the present study that could be addressed in future research. First, we did not collect information about participants' ethnicity or country of residence due to time restrictions imposed by the museum. Previous research has shown that culture can affect levels of prosocial behaviour (Trommsdorff, Friedlmeier, & Mayer, 2007) and the extent to which people are susceptible to social influence (Bond & Smith, 1996). Second, there are others factors that have previously been found to affect both prosocial behaviour and social influence, such as personality (e.g., Flynn, Ehrenreich, Beron, & Underwood, 2015) and friendship quality (Urberg, Luo, Pilgrim, & Degirmencioglu, 2003). Future research should seek to understand how these factors impact on the relationship between age and prosocial influence found in the current study.

We did not include any scenarios depicting non‐prosocial behaviour, such as neutral or antisocial behaviour. As a result, we cannot draw conclusions from the current study about whether the results are unique to prosocial behaviour (although studies indicate that age‐related decreases in social influence are seen for other types of behaviour too, such as risk perception and antisocial behaviour; Knoll et al., 2015, 2017; Steinberg & Monahan, 2007; Sumter et al., 2009). Further studies could assess how age affects prosocial, neutral and antisocial influence within the same paradigm. It is also possible that the museum context affected participants' ratings. For example, although the study took place in a secluded area, the participants could still hear noises from exhibits in the nearby gallery. The current study should be carried out in other settings to assess whether the results are replicated in different contexts.

A final limitation is that the present study asked participants to give hypothetical answers about how likely they would be to engage in prosocial behaviours, and did not assess prosocial behaviour directly. With moral behaviour like the prosocial behaviours described in the current study, there is some evidence of a discrepancy between what people report they will do and what they actually do (e.g., Teper, Inzlicht, & Page‐Gould, 2011). For example, there is evidence that children say they will give more in a Dictator Game than what they actually give (Blake, 2018). Another study showed that young adults kept slightly more money for themselves in a real versus hypothetical economic game in which a confederate receives electric shocks when the participant keeps money for themselves (FeldmanHall et al., 2012). Findings in this area are not entirely consistent: one study with adults found that the amount given in a Dictator Game with hypothetical money was very similar to the amount given in a game with real money (Ben‐Ner, Kramer, & Levy, 2008), although this relationship varied depending on the personality traits extraversion and agreeableness. To further understand age effects on susceptibility to prosocial influence, subsequent studies should use observational or experimental measures of actual prosocial behaviour such as charitable donations.

## 5 CONCLUSIONS

It has previously been established that susceptibility to social influence decreases from childhood to adulthood (e.g., Knoll et al., 2015). The current study found that the same age effects exist for prosocial influence, with children and adolescents more likely than adults to change their prosocial ratings as a result of social influence. The results demonstrate that young people's increased susceptibility to social influence can have a positive dimension, and should not exclusively be viewed in the context of risky or antisocial behaviour such as drug use and delinquency (van Hoorn, Fuligni, et al., 2016). The enhanced propensity for prosocial influence in children and adolescents could be harnessed when considering ways to promote prosocial behaviour in these age groups.

## References

[desc12666-bib-0001] Barry, C.M. , & Wentzel, K.R. (2006). Friend influence on prosocial behavior: The role of motivational factors and friendship characteristics. Developmental Psychology, 42, 153–163. 16420125 10.1037/0012-1649.42.1.15

[desc12666-bib-0002] Bates, D. , Mächler, M. , Bolker, B. , & Walker, S. (2014). Fitting linear mixed‐effects models using lme4. Journal of Statistical Software. Available at: http://arxiv.org/abs/1406.5823.

[desc12666-bib-0003] Batson, C.D. (1987). Prosocial motivation: Is it ever truly altruistic? In Berkowitz L. (Ed.), Advances in experimental social psychology (pp. 65–122). San Diego, CA: Academic Press.

[desc12666-bib-0004] Ben‐Ner, A. , Kramer, A. , & Levy, O. (2008). Economic and hypothetical dictator game experiments: Incentive effects at the individual level. Journal of Socio‐Economics, 37, 1775–1784.

[desc12666-bib-0005] Blake, P.R. (2018). Giving what one should: Explanations for the knowledge‐behavior gap for altruistic giving. Current Opinion in Psychology, 20, 1–5. 28822896 10.1016/j.copsyc.2017.07.041

[desc12666-bib-0006] Blakemore, S.‐J. , & Choudhury, S. (2006). Development of the adolescent brain: Implications for executive function and social cognition. Journal of Child Psychology and Psychiatry, 47, 296–312. 16492261 10.1111/j.1469-7610.2006.01611.x

[desc12666-bib-0007] Bond, R. , & Smith, P.B. (1996). Culture and conformity: A meta‐analysis of studies using Asch's (1952b, 1956) line judgment task. Psychological Bulletin, 119, 111–137.

[desc12666-bib-0008] Caouette, J.D. , & Ewing, S.W.F. (2017). Four mechanistic models of peer influence on adolescent cannabis use. Current Addiction Reports, 4, 90–99. 29104847 10.1007/s40429-017-0144-0 PMC5663303

[desc12666-bib-0009] Chein, J. , Albert, D. , O'Brien, L. , Uckert, K. , & Steinberg, L. (2011). Peers increase adolescent risk taking by enhancing activity in the brain's reward circuitry. Developmental Science, 14, F1–F10. 21499511 10.1111/j.1467-7687.2010.01035.x PMC3075496

[desc12666-bib-0010] Choukas‐Bradley, S. , Giletta, M. , Cohen, G.L. , & Prinstein, M.J. (2015). peer influence, peer status, and prosocial behavior: An experimental investigation of peer socialization of adolescents' intentions to volunteer. Journal of Youth and Adolescence, 44, 2197–2210. 26525387 10.1007/s10964-015-0373-2 PMC5985442

[desc12666-bib-0011] Dolan, L.J. , Kellam, S.G. , Brown, C.H. , Werthamer‐Larsson, L. , Rebok, G.W. , Mayer, L.S. , … Wheeler, L. (1993). The short‐term impact of two classroom‐based preventive interventions on aggressive and shy behaviors and poor achievement. Journal of Applied Developmental Psychology, 14, 317–345.

[desc12666-bib-0012] FeldmanHall, O. , Mobbs, D. , Evans, D. , Hiscox, L. , Navrady, L. , & Dalgleish, T. (2012). What we say and what we do: The relationship between real and hypothetical moral choices. Cognition, 123, 434–441. 22405924 10.1016/j.cognition.2012.02.001 PMC3355304

[desc12666-bib-0013] Flynn, E. , Ehrenreich, S.E. , Beron, K.J. , & Underwood, M.K. (2015). Prosocial behavior: Long‐term trajectories and psychosocial outcomes. Social Development, 24, 462–482. 26236108 10.1111/sode.12100 PMC4517683

[desc12666-bib-0014] Frey, B.S. , & Meier, S. (2004). Social comparisons and pro‐social behavior: Testing “conditional cooperation” in a field experiment. American Economic Review, 94, 1717–1722.

[desc12666-bib-0015] Gardner, M. , & Steinberg, L. (2005). Peer influence on risk taking, risk preference, and risky decision making in adolescence and adulthood: An experimental study. Developmental Psychology, 41, 625–635. 16060809 10.1037/0012-1649.41.4.625

[desc12666-bib-0016] Knoll, L.J. , Leung, J.T. , Foulkes, L. , & Blakemore, S.‐J. (2017). Age‐related differences in social influence on risk perception depend on the direction of influence. Journal of Adolescence, 60, 53–63. 28753485 10.1016/j.adolescence.2017.07.002 PMC5614112

[desc12666-bib-0017] Knoll, L.J. , Magis‐Weinberg, L. , Speekenbrink, M. , & Blakemore, S.‐J. (2015). Social influence on risk perception during adolescence. Psychological Science, 26, 583–592. 25810453 10.1177/0956797615569578 PMC4426139

[desc12666-bib-0018] Lam, C.B. , McHale, S.M. , & Crouter, A.C. (2014). Time with peers from middle childhood to late adolescence: Developmental course and adjustment correlates. Child Development, 85, 1677–1693. 24673293 10.1111/cdev.12235 PMC4107039

[desc12666-bib-0019] Larson, R. , & Richards, M.H. (1991). Daily companionship in late childhood and early adolescence: Changing developmental contexts. Child Development, 62, 284–300. 2055123 10.1111/j.1467-8624.1991.tb01531.x

[desc12666-bib-0020] Law, B.M.F. , Shek, D.T.L. , & Ma, C.M.S. (2013). Validation of family, school, and peer influence on volunteerism scale among adolescents. Research on Social Work Practice, 23, 458–466.

[desc12666-bib-0021] Lundborg, P. (2006). Having the wrong friends? Peer effects in adolescent substance use. Journal of Health Economics, 25, 214–233. 15964090 10.1016/j.jhealeco.2005.02.001

[desc12666-bib-0022] Masten, C.L. , Juvonen, J. , & Spatzier, A. (2009). Relative importance of parents and peers: Differences in academic and social behaviors at three grade levels spanning late childhood and early adolescence. Journal of Early Adolescence, 29, 773–799.

[desc12666-bib-0023] McAuliffe, K. , Raihani, N.J. , & Dunham, Y. (2017). Children are sensitive to norms of giving. Cognition, 167, 151–159. 28129890 10.1016/j.cognition.2017.01.006

[desc12666-bib-0024] Nelson, E.E. , Leibenluft, E. , McClure, E.B. , & Pine, D.S. (2005). The social re‐orientation of adolescence: A neuroscience perspective on the process and its relation to psychopathology. Psychological Medicine, 35, 163–174. 15841674 10.1017/s0033291704003915

[desc12666-bib-0025] Nook, E.C. , Ong, D.C. , Morelli, S.A. , Mitchell, J.P. , & Zaki, J. (2016). Prosocial conformity: Prosocial norms generalize across behavior and empathy. Personality and Social Psychology Bulletin, 42, 1045–1062. 27229679 10.1177/0146167216649932

[desc12666-bib-0026] O'Brien, S.F. , & Bierman, K.L. (1988). Conceptions and perceived influence of peer groups: interviews with preadolescents and adolescents. Child Development, 59, 1360–1365. 3168646 10.1111/j.1467-8624.1988.tb01504.x

[desc12666-bib-0027] Paluck, E.L. , Shepherd, H. , & Aronow, P.M. (2016). Changing climates of conflict: A social network experiment in 56 schools. Proceedings of the National Academy of Sciences, USA, 113, 566–571. 10.1073/pnas.1514483113 PMC4725542 26729884

[desc12666-bib-0028] Peysakhovich, A. , & Rand, D.G. (2015). Habits of virtue: Creating norms of cooperation and defection in the laboratory. Management Science, 62, 631–647.

[desc12666-bib-0029] R Core Team (2013). R: A language and environment for statistical computing. R Foundation for Statistical Computing, Vienna, Austria. URL http://www.R-project.org/.

[desc12666-bib-0030] Rushton, J.P. (1975). Generosity in children: Immediate and long‐term effects of modeling, preaching, and moral judgment. Journal of Personality and Social Psychology, 31, 459–466.

[desc12666-bib-0031] Schielzeth, H. , & Nakagawa, S. (2013). Nested by design: Model fitting and interpretation in a mixed model era. Methods in Ecology and Evolution, 4, 14–24.

[desc12666-bib-0032] Shang, J. , & Croson, R. (2009). A field experiment in charitable contribution: The impact of social information on the voluntary provision of public goods. Economic Journal, 119, 1422–1439.

[desc12666-bib-0033] Steinberg, L. , & Monahan, K.C. (2007). Age differences in resistance to peer influence. Developmental Psychology, 43, 1531–1543. 18020830 10.1037/0012-1649.43.6.1531 PMC2779518

[desc12666-bib-0034] Sumter, S.R. , Bokhorst, C.L. , Steinberg, L. , & Westenberg, P.M. (2009). The developmental pattern of resistance to peer influence in adolescence: Will the teenager ever be able to resist? Journal of Adolescence, 32, 1009–1021. 18992936 10.1016/j.adolescence.2008.08.010

[desc12666-bib-0035] Teper, R. , Inzlicht, M. , & Page‐Gould, E. (2011). Are we more moral than we think? Exploring the role of affect in moral behavior and moral forecasting. Psychological Science, 22, 553–558. 21415242 10.1177/0956797611402513

[desc12666-bib-0036] Trommsdorff, G. , Friedlmeier, W. , & Mayer, B. (2007). Sympathy, distress, and prosocial behavior of preschool children in four cultures. International Journal of Behavioral Development, 31, 284–293.

[desc12666-bib-0037] Urberg, K.A. , Luo, Q. , Pilgrim, C. , & Degirmencioglu, S.M. (2003). A two‐stage model of peer influence in adolescent substance use: Individual and relationship‐specific differences in susceptibility to influence. Addictive Behaviors, 28, 1243–1256. 12915166 10.1016/s0306-4603(02)00256-3

[desc12666-bib-0038] van den Bos, W. (2013). Neural mechanisms of social reorientation across adolescence. Journal of Neuroscience, 33, 13581–13582. 23966681 10.1523/JNEUROSCI.2667-13.2013 PMC6618652

[desc12666-bib-0039] van Goethem, A.A.J. , van Hoof, A. , van Aken, M.A.G. , Orobio de Castro, B. , & Raaijmakers, Q.A.W. (2014). Socialising adolescent volunteering: How important are parents and friends? Age dependent effects of parents and friends on adolescents' volunteering behaviours. Journal of Applied Developmental Psychology, 35, 94–101.

[desc12666-bib-0040] van Hoorn, J. , Fuligni, A.J. , Crone, E.A. , & Galván, A. (2016). Peer influence effects on risk‐taking and prosocial decision‐making in adolescence: Insights from neuroimaging studies. Current Opinion in Behavioral Sciences, 10, 59–64.

[desc12666-bib-0041] van Hoorn, J. , van Dijk, E. , Meuwese, R. , Rieffe, C. , & Crone, E.A. (2016). Peer influence on prosocial behavior in adolescence. Journal of Research on Adolescence, 26, 90–100.

