# Supplementary figures and images for: Age differences in the prosocial influence effect
Source: Dev Sci. 2018 Apr 15;21(6):e12666. doi: 10.1111/desc.12666 (PMC6221149; doi:10.1111/desc.12666)

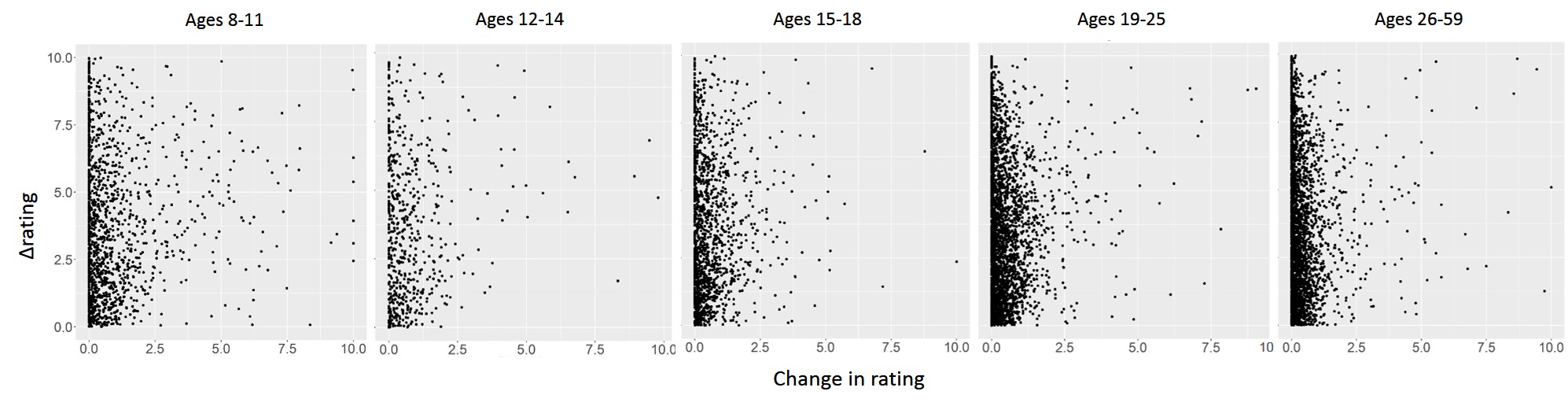

Supplement: Supplementary file 1 [file DESC-21-na-s001.jpg]

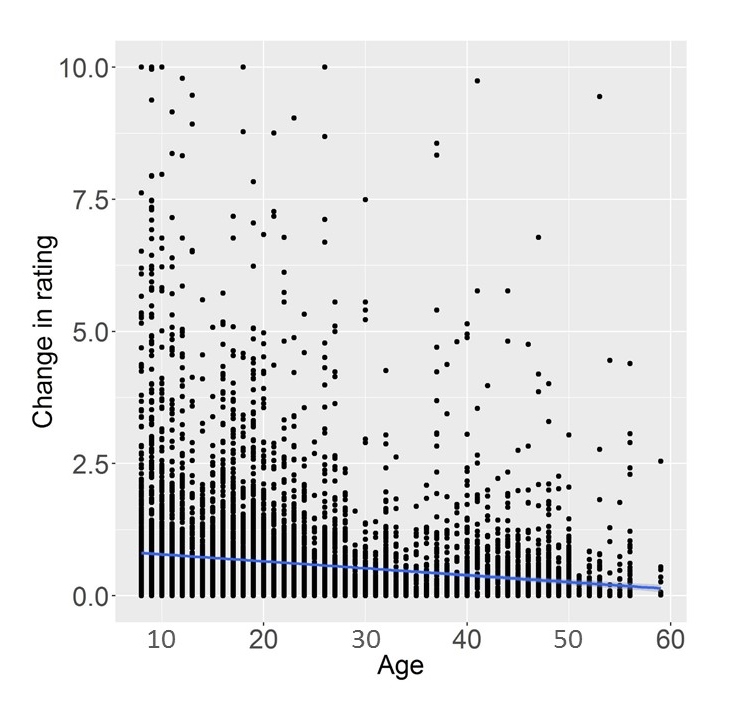

Supplement: Supplementary file 2 [file DESC-21-na-s002.jpg]
