# Supplementary material for: Age differences in the prosocial influence effect
Source: Dev Sci. 2018 Apr 15;21(6):e12666. doi: 10.1111/desc.12666 (PMC6221149; doi:10.1111/desc.12666)
Supplement: Supplementary file 3 [file DESC-21-na-s003.docx]

# **Supplementary Materials**

## **Method**

*Full list of prosocial behaviour scenarios*

Give up your time to help a friend

Give up your time to help a family member

Give up your time to help a neighbour

Visit a friend when they are ill

Visit a family member when they are ill

Visit a neighbour when they are ill

Care for a friend when they are ill

Care for a family member when they are ill

Care for a neighbour when they are ill

Give up your seat for a friend on the bus

Give up your seat for a family member on the bus

Give up your seat to a stranger on the bus

Give up your seat for a friend on the train

Give up your seat for a family member on the train

Give up your seat to a stranger on the train

Pick up something that a friend has dropped

Pick up something that a family member has dropped

Pick up something that a stranger has dropped

Carry a friend's bag for them

Carry a family member's bag for them

Make a friend a birthday card

Make a family member a birthday card

Buy a friend a birthday card

Buy a family member a birthday card

Stand up for a friend when they are being teased

Stand up for a family member when they are being teased

Stand up for a stranger when they are being teased

Stand up for a friend when they are being bullied

Stand up for a family member when they are being bullied

Stand up for a stranger when they are being bullied

Give something you like to charity

Help tidy up a friend's mess

Help tidy up a family member else’s mess

Lend a friend your favourite book

Lend a family member your favourite book

Lend a neighbour your favourite book

Let a friend go ahead of you in a queue

Let a family member go ahead of you in a queue

Let a stranger go ahead of you in a queue

Lend a friend your favourite clothes

Lend a family member your favourite clothes

Lend a neighbour your favourite clothes

Raise money for charity

Buy a friend a present

Buy a family member a present

Look after a friend's pet when they are away

Look after a family member's pet when they are away

Look after a neighbour's pet when they are away

Give money to charity

Show a friend where to go if they are lost

Show a family member where to go if they are lost

Show a stranger where to go if they are lost

Water a friend's plants when they are away

Water a family member's plants when they are away

Water a neighbour's plants when they are away

Feed a friend's pet when they are away

Feed a family member's pet when they are away

Feed a neighbour's pet when they are away

Take a friend to the nurse or doctor when they are ill

Make a friend a present

Make a family member a present

Help a friend clean their car

Help a family member clean their car

Help a neighbour clean their car

Help a friend clean their house

Help a family member clean their house

Help a neighbour clean their house

Lend a friend money

Lend a family member money

Lend a neighbour money

Volunteer for a charity

Help a friend if they have fallen

Help a family member if they have fallen

Help a stranger if they have fallen

Help a friend with their work

Help a family member with their work

Help a neighbour with their work

Babysit for a family member

Babysit for a neighbour

## **Results**

| Group A | Group B | Mean difference (A-B) | SE | Z ratio | p |
| --- | --- | --- | --- | --- | --- |
| Children | Young adolescents | .106 | .203 | .523 | 1.000 |
|  | Mid. adolescents | .042 | .159 | .261 | 1.000 |
|  | Young adults | -.030 | .140 | -.211 | 1.000 |
|  | Adults | -.278 | .139 | -1.997 | .459 |
| Young adolescents | Mid. adolescents | -.065 | .201 | -.322 | 1.000 |
|  | Young adults | -.136 | .187 | -.728 | 1.000 |
|  | Adults | -.384 | .184 | -2.089 | .367 |
| Mid. adolescents | Young adults | -.071 | .137 | -.520 | 1.000 |
|  | Adults | -.319 | .136 | -2.347 | .189 |
| Young adults | Adults | -.248 | .113 | -2.194 | .282 |

**Supplementary Table 1. Pairwise comparisons between age groups of rating 1. Bonferroni-corrected p-values are shown.**

N.B. Means (SD): children = 6.63 (.19); young adolescents = 6.51 (.11); mid. adolescents = 6.67 (.30); young adults = 6.68 (.19); adults = 6.98 (.48)

| Baseline group | Comparison group | t | DF | p |
| --- | --- | --- | --- | --- |
| Children | Young adolescents | -1.683 | 743 | .929 |
|  | Mid. adolescents | -5.451 | 718 | .000 |
|  | Young adults | -8.565 | 716 | .000 |
|  | Adults | -10.33 | 709 | .000 |
| Young adolescents | Mid. adolescents | -2.509 | 758 | .123 |
|  | Young adults | -4.417 | 763 | .000 |
|  | Adults | -5.685 | 773 | .000 |
| Mid. adolescents | Young adults | -2.384 | 747 | .174 |
|  | Adults | -4.117 | 740 | .000 |
| Young adults | Adults | -2.059 | 746 | .398 |

**Supplementary Table 2. Pairwise comparisons between age groups of change in rating score. Bonferroni-corrected p-values are shown.**

N.B. Slopes for Δrating (SD): children = .117 (.011); young adolescents = .085 (.016); mid. adolescents = .038 (.011); young adults = .008 (.008); adults = -.013 (.008)

**Prosocial influence analysis with age as a continuous variable**

We conducted an additional linear mixed effect model in which age was included as a continuous variable. The model was otherwise the same as the main prosocial influence analysis, and can be represented as follows:

**change in rating = Δrating + (Δrating x continuous age) + (Δrating x source) + (Δrating x source x continuous age)**

The results showed a significant main effect of Δrating (χ^2^(1) =26.79, *p* <.001), indicating that participants demonstrated greater changes from rating 1 to rating 2 when the difference between their first rating and provided rating was greater. This is in line with the categorical age analysis.

There was a significant interaction between Δrating and age as a continuous variable (χ^2^(1) = 80.33, *p*<.001), indicating that the age of participants affected the extent to which they were socially influenced. Specifically, as age increased, their susceptibility to prosocial influence decreased. This is also in line with the categorical age analysis.

There was no significant interaction between Δrating and source type (χ^2^(1) =2.14, *p* = .144), and no three-way interaction between Δrating, source type and age as a continuous variable (χ^2^(1) =.16, *p* =.687), indicating that the source of information (adolescent or adult) did not affect the extent to which participants were socially influenced. This was also the case in the categorical age analysis.

To illustrate this linear decrease across age, we have plotted change in rating (absolute difference between rating 1 and rating 2) against age in Supplementary Figure 2.
